# Novel de novo dominant *PSMB10* variants in three patients with immune deficiency and liver disease

**DOI:** 10.70962/jhi.20250096

**Published:** 2025-11-18

**Authors:** Benjamin Fournier, Léa Poirier, Vincent Abramowski, Etienne Merlin, Hélène Deutsch, Paul Bastard, Isabelle Callebaut, Capucine Picard, Jérémie Rosain, Sébastien Küry, Stéphane Bézieau, Monique Fabre, Martin Castelle, Bénédicte Neven, Despina Moshous, Jean-Pierre de Villartay, Frédéric Ebstein

**Affiliations:** 1 Paediatric Haematology-Immunology and Rheumatology Unit, Necker Hospital, AP-HP.Centre - Université Paris Cité, Paris, France; 2 Laboratory of Lymphocyte Activation and Susceptibility to EBV Infection, Inserm UMR 1163, Institut Imagine, Paris, France; 3 Nantes Université, CNRS, INSERM, l’institut du Thorax, Nantes, France; 4 Université Paris Cité, Imagine Institute, Laboratory “Genome Dynamics in the Immune System,” INSERM UMR 1163, Paris, France; 5 Equipe Labellisée Ligue Nationale Contre le Cancer, Paris, France; 6 Department of Pediatrics, Clermont-Ferrand University Hospital, Clermont-Ferrand, France; 7 Clermont Auvergne University, INSERM, Clermont-Ferrand, France; 8 Clermont Auvergne University, INRA, UMR 1019 UNH, ECREIN, Clermont-Ferrand, France; 9 Department of Pediatric Onco-Hematology, University Hospital, Vandoeuvre-les-Nancy, France; 10 Laboratory of Human Genetics of Infectious Diseases, Necker Branch, INSERM U1163, Paris, France; 11 Paris Cité University, Imagine Institute, Paris, France; 12 St. Giles Laboratory of Human Genetics of Infectious Diseases, Rockefeller Branch, The Rockefeller University, New York, NY, USA; 13 Sorbonne Université, Muséum National d'Histoire Naturelle, UMR CNRS 7590, Institut de Minéralogie, de Physique des Matériaux et de Cosmochimie, IMPMC, Paris, France; 14 Study Center for Primary Immunodeficiencies, Necker Hospital, AP-HP.Centre - Université Paris Cité, Paris, France; 15 Nantes Université, CHU de Nantes, Service de Génétique Médicale, Nantes, France; 16 Department of Pathology, Necker Hospital, AP-HP.Centre - Université Paris Cité, Paris, France; 17 Laboratory of Immunogenetics of Pediatric Autoimmune Diseases, Université Paris Cité, Imagine Institut, INSERM UMR 1163, Paris, France; 18 Centre de référence des déficits Immunitaires Héréditaires, Necker Hospital, AP-HP.Centre - Université Paris Cité, Paris, France

## Abstract

Background and objective: Despite significant advances, the molecular basis and thus patient-tailored therapeutic options for inborn errors of immunity remain unknown in a significant number of patients. Methods: We investigated three patients from unrelated families with inborn errors of immunity and severe liver disease. Clinical, immunological, and histological (only in one patient for the latter) data were collected, and trio whole exome sequencing was performed. Results: Whole exome sequencing identified heterozygous de novo variants in *PSMB10* (p.Asp205Ala and p.Ser208Phe), a gene encoding a specific subunit of the immuno- and thymoproteasome. Analysis showed poor integration into the proteasome complex of PSMB10 variants, thereby exerting a dominant-negative effect on the PSMB9 subunit. Conclusion: Dominant *PSMB10* variants should be considered in genetic screening for SCID and CID, especially in patients with associated liver disease. Very severe endothelial-like disease before and after HSCT and very poor outcomes after HSCT should weigh up the indication of HSCT.

## Teaser

Novel dominant PSMB10 variants cause severe combined immunodeficiency and life-threatening liver disease in three pediatric patients. These mutations impair immunoproteasome assembly through a dominant-negative effect on PSMB9, likely disrupting lymphocyte survival and endothelial function, and are associated with poor outcomes after hematopoietic stem cell transplantation.

## Introduction

Genetic defects that impair T cell development in the bone marrow or in the thymus can cause severe combined immune deficiencies (SCID) or combined immune deficiencies (CID).

These defects may either directly affect thymic epithelial cells (TECs) or disrupt the cellular machinery involved in the survival, proliferation, and differentiation of immature T cells/thymocytes (1).

The proteasome complex is essential for cell survival and proliferation and represents a protease system responsible for the regulated degradation of intracellular proteins, primarily those tagged with ubiquitin. The 20S core particle (CP) forms the catalytic heart of the proteasome, consisting of four stacked heptameric rings. The outer α-rings regulate substrate entry through gated pores formed by N-terminal residues, while the inner β-rings contain three catalytic subunits (PSMB6, PSMB7, and PSMB5) with distinct cleavage specificities: caspase-like, trypsin-like, and chymotrypsin-like activities, respectively. Assembly of the 20S CP requires specialized chaperones (PAC1–4 and POMP) that guide α-ring formation, β-subunit incorporation, and pro-peptide processing. The fully functional 26S proteasome is formed by the 20S CP capped at one or both ends with 19S regulatory particles (RPs), with each 19S RP recognizing substrates, unfolding them, and translocating them into the 20S catalytic chamber. Alternative regulators like PA28, composed of PSME1 and PSME2 subunits, can replace one 19S particle at one end of the 20S CP to form hybrid complexes, enhancing peptide hydrolysis and participating in immune antigen processing (2).

The proteasome relies on the assembly of the core subunits PSMB1-4, common to all cell types, and the addition of cell-specific ones: PSMB5, PSMB6, and PSMB7 for the constitutive proteasome in most cell types (except lymphocytes and TEC), PSMB8, PSMB9, and PSMB10 for the immunoproteasome, PSMB9, PSMB10, and PSMB11 in TEC.

Pathogenic mutations in components of the proteasome are responsible for a rare autoinflammatory condition related to interferonopathies called proteasome-associated autoinflammatory syndrome (PRAAS) (3). While PRAAS is primarily caused by inherited biallelic mutations in one of the PSMBx subunits, two patients with a distinctive autoinflammatory phenotype have been reported with de novo heterozygous mutations in *PSMB9* (4).

We report three patients harboring hitherto de novo heterozygous variants in proteasome 20S subunit β 10 (PSMB10) encoding a subunit of only in the immuno- and thymoproteasome. Their clinical phenotype and propensity for liver disease were strongly reminiscent of recently reported cases of patients with other *PSMB10* variations (5, 6), further emphasizing the risk of severe endothelial disease, hence questioning the advisability of hematopoietic stem cell transplantation (HSCT).

## Results

### Three patients with combined immunodeficiency and severe liver disease

Patient I (PI) was a girl born from unrelated Caucasian parents. She presented at the age of 3 mo *Pneumocystis jirovecii*–induced hypoxic pneumonia and was diagnosed with T-B-NK+ SCID. At 5 mo of age, she received haploidentical HSCT preceded by a myeloablative reduced-toxicity conditioning regimen (CR) with busulfan (measured total area under the curve [AUC] 68,940 ng/mLxh, fludarabine 160 mg/m^2^, and thymoglobulin 7.5 mg/kg), as recommended for SCID (7). Liver enzymes and ultrasound were normal before transplant except for a mild homogeneous hepatomegaly; nonetheless, she received prophylactic defibrotide due to her young age. She developed early and fulminant veno-occlusive disease (VOD) (day [D]+3 after HSCT) with liver failure and systemic inflammatory response requiring several weeks of intensive care. She died at D+66 after HSCT from *Pseudomonas aeruginosa* sepsis. She had no clinical signs suggesting a DNA repair-deficiency disorder (i.e., normal milestones, normal head circumference, height, and weight).

**Figure 1.**
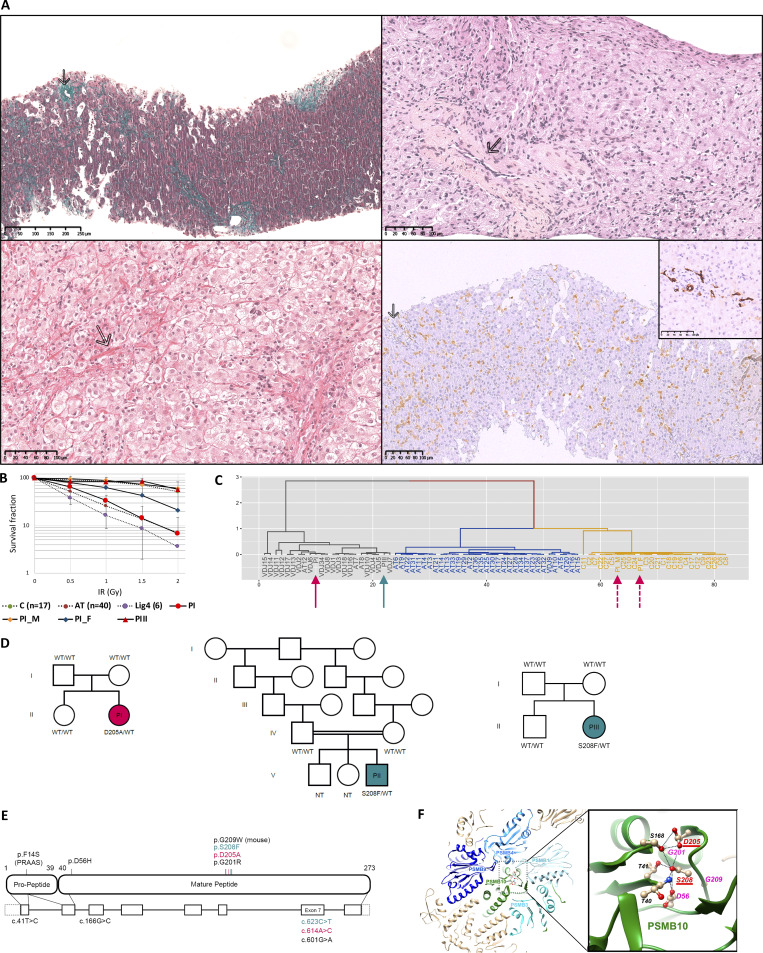
***PSMB10* variants with biological and histological features. (A)** Liver histology of PII. Left upper part: Partial phlebosclerosis of central vein (arrow) and sinusoidal dilatation with aggregation of erythrocytes and lymphocytes, trichrome staining, ×13. Right upper part: Chronic disease, with shrunken other central vein and fibrous occlusion (arrow), binucleation and hepatocyte anisonucleosis, HES staining, ×20. Left lower part: Zone 3 perisinusoidal fibrosis (arrow) with loss or atrophy of hepatocytes, associated with areas of hepatocyte regeneration (plump hepatocytes with pale cytoplasm), no nodular regenerative hyperplasia, Sirius red staining, ×22. Right lower part: Very mild portal inflammation (arrow) and moderate lobular inflammation with CD3^+^, CD8^+^ in sinusoids, CD3 immunostaining, ×16. Inset on the right lower part. Detail of a near normal portal tract in the insert, cytokeratin 7 immunostaining, ×25. **(B)** Radiosensitivity testing of PBMC of PI, PI’s mother and father (PI_M and PI_F), and PIII compared to healthy controls (C, green), ataxia telangiectasia (AT, brown), and DNA-ligaseIV (Lig4, purple) radiosensitive patients. No fibroblast available for all patients. **(C)** PROMIDISα signature clustering of PI (red arrow), PI’s mother and father (PI_M and PI_F, red dotted arrows), PIII (green arrow), and group of V(D)J/DNA repair-deficient patients (grey), patients with ataxia telangiectasia (blue), and healthy control individuals (yellow). **(D)** Family pedigrees for PI, PII and PIII. **(E)** Schematic representation of *PSMB10* intron–exon organization (intron in lines and coding regions in boxes, lower part) with corresponding protein (upper part), respectively, and noncoding exon sequences (in dashed boxes). Variants of the three patients are colored; the PRAAS biallelic mutation, other *PSMB10* heterozygous mutations involved in inborn errors of immunity, and the TUB6 mouse mutation are represented in black. **(F)** Cryo-EM 3D structure of human PSMB10 within the immunoproteasome complex, with positions of mutated amino acids labeled in red (this study) and pink (Protein Data Bank accession no. 6AVO [8]). H-bonds are shown with broken lines. WT, wild-type.

PII was a consanguineous boy born from North African parents. He presented with severe diarrhea starting from 15 days of life requiring parenteral feeding for several weeks. At 2 mo of age, he developed a severe liver disease resembling VOD with recurrent flares of edematous liver failure and esophageal varices, with CMV replication at first episode but no CMV disease. Liver histology was strongly indicative of VOD without CMV staining (sinusoidal dilatation, perisinusoidal fibrosis, and fibrous occlusion of central veins, Fig. 1 A). Digestive tract histology showed total villous atrophy with inflammation at 7 mo of age (M7) and villous regeneration with B and T cell infiltrates at M12. Comprehensive microbiological, autoimmune and metabolic workups were negative. He had thrombotic microangiopathy in the first year of life with proteinuria, hypertension, and schizocytes requiring platelet and erythrocyte transfusions.

After several lines of immunosuppressive treatments, including corticosteroids, sirolimus, and cyclosporin A, parenteral and then enteral feeding could be weaned off, and the liver disease stabilized to moderate cytolysis. He unexpectedly died of what appeared to be a flare of liver VOD.

Patient III (PIII) was a girl born from unrelated Caucasian parents, who presented with *P. jirovecii*–induced hypoxic pneumonia at 3 mo of age with lymphopenia but normal naïve T cell count, severe chronic diarrhea from 4 mo of age requiring enteral feeding and severe atopic dermatitis, which evolved into erythroderma at 7 mo of age.

At 8 mo of age, she had primary CMV infection, which led to fulminant liver disease consistent with VOD with refractory ascites, refractory thrombocytopenia, and liver failure, leading to death.

In all patients, fever occurred with an expected increased CRP level range only in the context of infection or VOD flares.

Immune parameters were altered mostly in PI and PII with T cell lymphopenia, very low naive T cells, low percentage of TCR-Vα7.2-expressing T lymphocytes, and low B cells. In PII, T cell lymphopenia partially improved over time for CD4 T cells and completely recovered for CD8 T cells, while percentage of naïve CD4 and CD8 T cells remained low. By contrast, PIII presented with global lymphopenia but normal naïve T cell count, percentage of Vα7.2 lymphocytes, and B cell count. IgG levels were very low in PI and moderately decreased in PII and PIII, with PII requiring IVIG supplementation only until 9 mo of age. Complete immune workup is shown in Table 1.

**Table 1.** Immunological phenotype and additional epidemiological and clinical features

​	PI (3-mo-old)	PII (2-mo-old)	PIII (5-mo-old)	Age-matched normal values (0–6 mo)
IgG (g/L)	<1.08	1.76	1.84	2–12
IgA (g/L)	0.51	0.21	0.39	0.8–0.9
IgM (g/L)	0.07	0.11	0.28	0.1–0.9
CD3^+^ (/μl)	151	1,318	1,757	2,500–6,000
TCRγδ/CD3^+^	NP	10	1.8	​
CD4^+^ (/μl)	110	301	1,384	1,300–4,000
CD8^+^ (/μl)	24	994	340	400–1,900
CD31^+^CD45RA^+^/CD4^+^: naive (%)	2	0	52	90–95
CCR7^+^CD45RA^+^/CD8^+^: naive (%)	1	1	85	78–97
CCR7^+^CD45RA^−^/CD8^+^: central memory (%)	3	1	4.5	3–15
CCR7^−^CD45RA^−^/CD8^+^: effector memory (%)	63	68	4.9	0–3
CCR7^−^CD45RA^+^/CD8^+^: TEMRA (%)	33	30	5.1	0.1–7
CD19^+^ (/μl)	24	58	269	9,600-3,700
CD27^+^IgD^+^/CD19^+^: switched-memory B cells (%)	NP	11.5	6.1	0.1–1
CD16^+^CD56^+^ (/μl)	20	936	505	150–950
TCR Vα7.2/CD3^+^ (%)	0.5	0.5	2	1.3–7.3
Vβ repertoire/CD8^+^	NP	Mildly biased	Normal	​
Type-1 interferon signature	NP	NP	Normal	​
PHA-induced T cell proliferation	NP	Normal	Normal	​

NP: not performed.

Cell survival after ionizing radiations of pre-HSCT PBLs was significantly decreased in PI (Fig. 1 B) and comparable to that of ataxia-telangiectasia and Lig4 deficient patients (9) and was normal in PIII.

The PROMIDISα signature of PI pre-HSCT T cells and of PIII (methods reported in [10]) clustered with that of V(D)J/DNA repair-deficient patients, as opposed to both PI’s parents who presented a wild-type signature (Fig.1 C).

Trio-based whole exome sequencing (WES) in PI and PII targeted gene panel sequencings in PIII with familial segregation revealed de novo private heterozygous variants in *PSMB10*: c.614A>C p.Asp205Ala for PI and c.623C>T p.Ser208Phe for PII and PIII (Fig. 1 D), which were absent from gnomAD v4 and characterized by a AlphaMissense score of pathogenicity of 0.92 and 0.90, respectively (11). These residues are part of a conserved domain and participate in a bond network critical for PSMB10 internal cohesion and interaction with other proteasomes subunits (Fig. 1, E and F).

PSMB10 is part of the immunoproteasome in lymphocytes and the thymoproteasome in TECs. A SCID mouse (TUB6 mouse) carries a mutation in *Psmb10*, p.Gly170Trp (paralogous to human p.Gly209Trp) (12). Moreover, *PSMB10 *de novo variants have been reported very recently in patients with similar clinical presentations, affecting either an amino acid near residues 205 and 208 (p.Gly201Arg) or an amino acid predicted to interact with p.Ser208 (p.Asp56His) (Fig. 1, E and F) (5, 6).

### p.Asp205Ala and p.Ser208Phe *PSMB10* variants impair proteasome assembly and exert dominant-negative effects on PSMB9 expression

**Figure 2.**
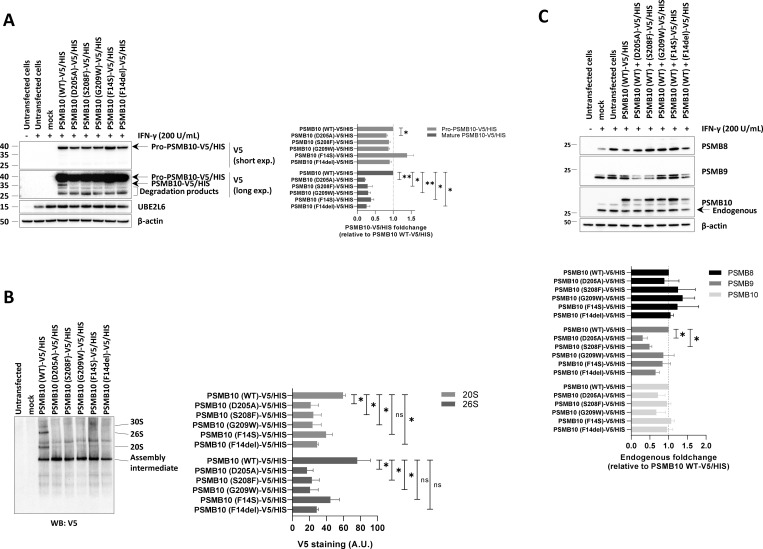
**Expression and incorporation of V5/HIS-tagged *PSMB10* single variants into proteasome complexes in HEK293T cells. (A)** HEK293T cells were treated with IFN-γ (200 U/ml) for 24 h, followed by transfection with various C-terminally V5/HIS-tagged *PSMB10* constructs. Proteins were extracted and analyzed by SDS-PAGE and western blotting using antibodies specific for V5, UBE2L6, and β-actin (loading control), as indicated. For V5 immunostaining, both short and long exposure times are shown. The immature (32.627 kDa) and mature (28.339 kDa) forms of PSMB10 protein (β2i) are marked with arrows, with the mature form visible only under long exposure. Representative data from one experiment are shown. The right panel displays densitometric analyses of V5 immunoreactive bands. Data are presented as fold-change mean values ± SEM for each variant relative to their wild-type counterpart (set to 1, indicated by the gridline) from three independent experiments. Statistical significance was assessed using a paired *t* test (*P < 0.05, **P < 0.01). **(B)** Protein samples described in A were analyzed by native-PAGE, followed by western blotting using a V5-specific antibody. Arrows indicate the positions of the 30S, 26S, 20S, and assembly intermediate proteasome complexes. The right panel shows densitometric analyses of V5 immunoreactive bands. Data are presented as band intensity values ± SEM from three independent experiments. Statistical significance was assessed using a paired *t* test (*P < 0.05, **P < 0.01). **(C)** HEK293T cells were treated with IFN-γ (200 U/ml) for 24 h and subsequently transfected with various C-terminally V5/HIS-tagged PSMB10 constructs. Protein lysates were extracted and analyzed by SDS-PAGE, followed by western blotting using antibodies specific for the immunoproteasome subunits PSMB8, PSMB9, and PSMB10, as indicated. β-actin was used as a loading control to ensure equal protein loading. The right panel presents densitometric analyses of PSMB8, PSMB9, and PSMB10 immunoreactive bands, with data expressed as fold-change mean values ± SEM for each variant relative to the PSMB10 (WT)-V5/HIS condition (normalized to 1, as indicated by the gridline) across three independent experiments. Statistical significance was determined using a paired *t* test (*P < 0.05, **P < 0.01). WT, wild-type. Source data are available for this figure: SourceData F2.

**Figure S1.**
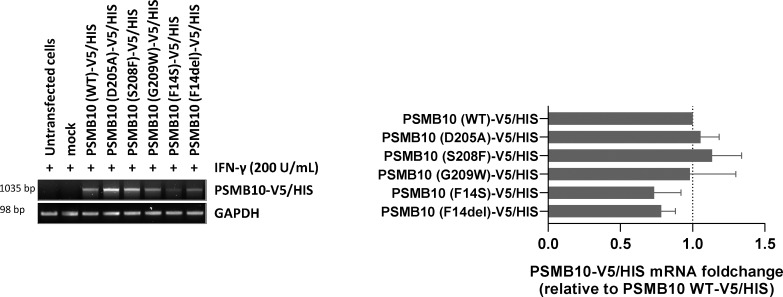
**mRNA expression levels of V5/HIS-tagged *PSMB10* variants in transfected HEK293T cells.** HEK293T cells transfected with various PSMB10-V5/HIS constructs were subjected to total RNA extraction, followed by semiquantitative RT-PCR using primers targeting *PSMB10* and the polyadenylation signal of the expression vector (BGH). GAPDH amplification was used as a loading control to ensure equal RNA input across samples. Right panel: Densitometric quantification of PSMB10-V5/HIS transcript levels. Data are presented as fold changes relative to wild-type (WT) PSMB10-V5/HIS, normalized to GAPDH, with WT levels set to 1 (indicated by the gridline). Values represent mean ± SEM from three independent experiments. Source data are available for this figure: SourceData FS1.

**Figure S2.**
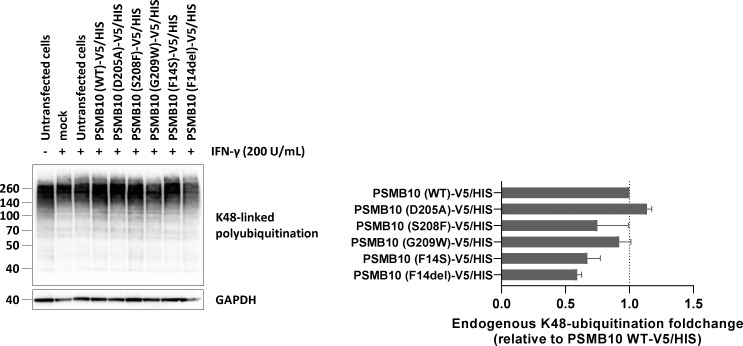
**Analysis of K48-linked ubiquitination profiles in HEK293T cells transfected with V5/HIS-tagged *PSMB10* variants.** HEK293T cells pretreated with IFN-γ (200 U/ml for 24 h) were transfected with various PSMB10-V5/HIS constructs and analyzed for K48-linked ubiquitin-protein conjugates by SDS-PAGE, followed by western blotting. GAPDH was used as a loading control to ensure equal protein input. Right panel: Densitometric quantification of high molecular weight (>70 kDa) ubiquitinated proteins. Data are shown as fold changes relative to wild-type (WT) PSMB10-V5/HIS, normalized to GAPDH, with WT levels set to 1 (indicated by the gridline). Results represent mean ± SEM from three independent experiments. Source data are available for this figure: SourceData FS2.

To assess the impact of p.Asp205Ala and p.Ser208Phe *PSMB10* variants on PSMB10 subunit expression and its subsequent incorporation into proteasomes, we expressed N-terminally V5/HIS-tagged wild-type and mutant *PSMB10* constructs in HEK293T cells prior to SDS-PAGE/western blotting analysis. Since PSMB10 assembly requires the coordinated incorporation of the immunoproteasome subunits PSMB8 and PSMB9 (13, 14), HEK293T cells were pretreated with IFN-γ to induce the endogenous upregulation of these subunits prior to transfection, as previously described (15). IFN-γ stimulation was effective, as evidenced by UBE2L6 upregulation (Fig. 2 A). Compared to wild-type PSMB10, both the p.Asp205Ala and, to a lesser extent, the p.Ser208Phe variants showed reduced steady-state levels of the PSMB10 precursor form (Fig. 2 A). Since semiquantitative RT-PCR revealed comparable PSMB10 mRNA levels across all constructs (Fig. S1), this reduction is likely due to a detrimental effect of the variants on PSMB10 protein stability rather than on transcript abundance. In addition, the p.Asp205Ala and p.Ser208Phe variants were associated with a complete absence of the mature PSMB10 subunit, to a degree comparable to that observed for the other three *PSMB10* variants, suggesting impaired subunit processing. Native-PAGE and western blot analysis further demonstrated a reduced content of V5-containing 20S, 26S, and 30S proteasome complexes in cells expressing any of the V5/HIS-tagged p.Asp205Ala, p.Ser209Phe, p.Gly209Trp, p.Phe14Ser, or p.Phe14del *PSMB10* variants compared to their wild-type counterpart (Fig. 2 B). Since these variants were identified in heterozygous patients, we investigated their potential dominant-negative effects on other immunoproteasome subunits. IFN-γ treatment upregulated endogenous PSMB8, PSMB9, and PSMB10 in mock-transfected cells (Fig. 2 C). However, despite comparable levels of endogenous PSMB10 and ubiquitin-protein conjugates, cells expressing either p.Asp205Ala or p.Ser208Phe *PSMB10* exhibited reduced endogenous PSMB9 levels (Fig. 2 B and Fig. S2), indicative of a dominant-negative effect. In contrast, this effect was observed neither with p.Phe14Ser nor with p.Phe14del variants previously associated with recessive inheritance (15, 16).

### p.Ser208Phe *PSMB10* variant reduces proteasome activity and hybrid proteasome complex levels in patient-derived T cells

**Figure 3.**
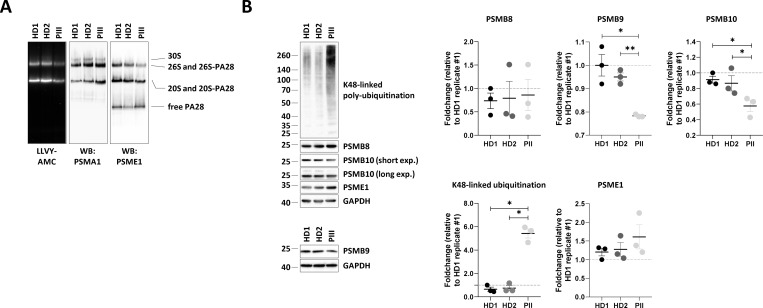
***PSMB10* p.Ser208Phe variant is associated with decreased immunoproteasome expression, reduced PA28–proteasome complexes, diminished proteasome activity, and disrupted protein homeostasis in PIII-derived T cells. (A)** T cells expanded from PBMCs of patient PIII and two age-matched healthy male and female donors (HD) were subjected to protein extraction, followed by native-PAGE separation and in-gel overlay assays (LLVY-AMC) to assess proteasome activity, as well as western blotting using PSMA1- and PSME1-specific antibodies. Lines indicate the migration positions of the 30S, 26S, and 26S–PA28 (hybrid) complexes, as well as the 20S and 20S–PA28 complexes, and the free PA28 heptameric ring. **(B)** Protein lysates from A were analyzed by SDS-PAGE and western blotting using antibodies directed to K48-linked ubiquitinated proteins PSMB8, PSMB9, PSMB10, PSME1, and GAPDH, with GAPDH serving as a loading control. Shown is one representative experiment (left) and densitometric analyses (right), with data expressed as fold-change mean values (*n* = 3) relative to a replicate of HD1, which was set to 1 after normalization to GAPDH. Statistical significance was assessed using a paired *t* test (*P < 0.05 and **P < 0.01). Source data are available for this figure: SourceData F3.

To corroborate the detrimental effects of the p.Ser208Phe *PSMB10* variant observed in vitro with in vivo evidence, we assessed proteasome function in T cells derived from PIII. In PIII’s T cells, gel-overlay assays showed significantly reduced chymotrypsin-like proteasome activity compared to age-matched controls (Fig. 3 A), which was not due to a lack of total proteasomes, as indicated by increased PSMA1 levels. Western blot analysis revealed a marked decrease in PA28-containing hybrid proteasomes (19S-20S-PA28 and 20S-PA28), suggesting that the p.Ser208Phe variant impaired the recruitment of PA28 to 20 and 26S proteasomes (Fig. 3 A). This defect correlated with an accumulation of ubiquitin-modified proteins, further supporting impaired proteasome-mediated protein breakdown (Fig. 3 B). Consistent with our overexpression experiments, patient-derived T cells exhibited ∼40% reduction in mature PSMB10 and a ∼20% decrease in PSMB9, while PSMB8 levels remained unchanged (Fig. 3 B).

## Discussion

*PSBM10* heterozygous variant is a newly reported cause of inborn errors of immunity (5, 6). We report three additional patients with a severe phenotype associated to new *PSMB10* variants that impair the immunoproteasome assembly.

The seven reported patients and the three patients reported here who harbor dominant *PSMB10* variants exhibited remarkably similar clinical phenotypes, characterized by skin, digestive tract, or liver involvements, *Pneumocystis*, and CMV infections (Table 2). Three patients experienced severe liver involvement prior to HSCT, in which CMV was considered a trigger rather than a cause: liver disease remained active despite CMV control in two, no CMV staining was documented in a liver biopsy in one (PII), and the context of liver failure in PIII resembled VOD more than CMV hepatitis.

**Table 2.** Summary of the 10 patients reported with *PSMB10* heterozygous de novo variations

​	PI	PII	PIII	P4	P5	P6	P7	P8	P9	P10
Variant	p.Asp205Ala	p.Ser208Phe	p.Ser208Phe	p.Gly201Arg	p.Gly201Arg	p.Gly201Arg	p.Gly201Arg	p.Asp56His	p.Gly201Arg	p.Gly201Arg
Reported in	Present report	Present report	Present report	(5)	(5)	(5)	(5)	(5)	(5)	(6)
**Clinical features**										
Enteropathy (age at onset)	No	Chronic diarrhea with transient TPN (2 wk)^a^^,^^b^	Chronic diarrhea (8 wk)^a^	Chronic diarrhea (8 wk)	Chronic diarrhea with TPN (colitis, no CMV) (1 mo)	Chronic diarrhea (3 wk)	Chronic diarrhea (6 wk)	Chronic diarrhea with TPN (birth)	Blood and mucus in stools (8 wk)	Chronic diarrhea (8 mo)
Liver disease	No	**VOD with multiple flares of liver failure, cholestasis, cytolysis, and esophageal varices** (2 mo)	**Liver failure with ascites** (8 mo)	NR	**Chronic hepatocellular and cholestatic liver failure** (1 mo)	NR	NR	NR	NR	Transaminitis (10 mo) and elevated liver function tests (13 mo)
TMA	No	**Yes** (1 year)	No	NR	NR	NR	NR	NR	NR	NR
Skin	No	No	Generalized erythroderma with desquamation (7 mo)	Generalized erythroderma with desquamation (1 wk)	Moderate facial erythroderma (2 wk)	Generalized erythroderma with desquamation (3 wk)	Generalized erythroderma with desquamation (1 wk)	Generalized maculopapular rash (8 wk)	Generalized dry skin (8 wk)	Pruritic rash (8 mo)
Infection	*Pneumocystis* pneumonia	Primary CMV infection at first VOD flare	*Pneumocystis* pneumonia	Oral candidiasis	CMV disseminated disease and encephalitis (1 mo)	Oral candidiasis	*Pneumocystis* in BAL (3 mo)	Adenovirus detected in blood and stools	No	*Pneumocystis* pneumonia
		Four catheter-related bacteremia	Primary CMV infection at liver failure				Oral candidiasis			Parainfluenza 3 respiratory infection (9 mo)
**HSCT characteristics**										
Age at HSCT	5 mo	NA	NA	2.8 mo	2.5 years	2.5 mo	16 wk	2.8 mo	2 mo	2.8 mo
Donor	MMRD	NA	NA	MSD	MRD	URD cord blood	MMRD	Mismatched cord blood	MMRD	MSD
Conditioning regimen	MAC-reduced toxicity^c^ (Bu, Flu, and ATG)	NA	NA	RIC (Cy and ATG)	RIC (Cy and ATG)	RIC (Mel and Flu)	MAC (Bu, Cy, and ATG)	RIC (Treo and Flu Alem)	RIC (Treo, Flu, ATG, and Rx)	RIC (Cy and ATG)
**Complications after HSCT**										
​	**Early and fulminant VOD (fatal)**; *P. aeruginosa* sepsis	​	​	***Encephalopathy, quadriplegia, neurogenic bladder*** (Csa toxicity) (Y3); myocarditis and dilated ***cardiomyopathy**** *after P B19 infection (Y4); adulthood: Skin hypersensitivity	**TMA (fatal)**	Skin GVHD; **severe VOD; chronic liver disease with cirrhosis and varices (sinusoidal obstruction syndrome);** ***chronic enteropathy; glomerulonephritis (mid-childhood) requiring kidney transplant***	**Capillary leak syndrome and pneumonitis**; GVHD skin and gut; ***fatal encephalopathy*** and VZV recurrence	Severe mucositis; skin toxicity; **liver dysfunction (histology: marked ductular cholestasis);** ***chronic enteropathy*** with norovirus infection; ***relapsing-remitting skin rash; profound and severe hypothyroidism***	Severe acute encephalopathy (M1, diffuse white matter involvement on MRI)	​
**Outcome**										
Outcome	Deceased (VOD and sepsis, 7 mo)	Deceased (edematous flare of VOD, 2 years)	Deceased (liver failure and with ascites, 8 mo)	Alive (18 years)	Deceased (TMA, 2 years)	Deceased (sepsis after kidney transplantation, 16 years)	Deceased (encephalopathy, 11 wk)	Deceased (sepsis, 4 years)	Alive (1 year)	Alive (>13 mo)
**Immune parameters**										
T cells	Low	Low	Low	Low	Low	Low	Low	Low	Low	Low
	No naïve T cells	No naïve T cells	Normal naïve CD8	Mildly decreased naïve CD4	No naïve T cell	No naïve T cells	No naïve T cells	No naïve T cells	No naïve T cells	Naïve T cells: normal at 5 and 8 mo; decreased at 14 mo
TREC	NP	NP	NP	NR	Low	NR	NR	NR	Low	Normal (Birth)
T cell repertoire diversity	NP	Reduced	Normal	Reduced	Reduced	Reduced	NR	NR	Normal	Reduced
B cells	Very low	Very low	Normal	Very low (Near complete medullar block)	Very low	Subnormal	Very low	Very low (Near complete medullar block)	Very low	Very low
NK cells	Low	Normal	Normal	Low	High	Low	Normal	Low	Normal	Low
Eosinophilia	Present	Absent	Present	Present	Present	Present	Present	Absent	Present	Present
T cell Proliferation	NP	PHA: normal	PHA: normal	PHA: reduced	PHA: reduced	PHA: reduced	PHA: reduced	PHA: reduced	PHA: reduced	PHA: normal
		OKT3: normal						OKT3: reduced	OKT3: normal	
IgGAM levels	Decreased IgGAM	Decreased IgGAM	Decreased IgGA	Decreased IgGAM	Decreased IgGAM	Decreased IgGA	Decreased IgG	Normal	Decreased IgGAM	Normal
Radiation sensitivity	Decreased (PBMC)	NP	Normal (PBMC)	Normal (fibroblast)	NR	NR	NR	NR	NR	NR
Type-1 interferon signature	NP	NP	Normal	NR	NR	NR	NR	NR	Normal	NR

Manifestations associated or very likely-associated to endothelial disease are bolded. Severe manifestations that persisted after HSCT with no argument for GVHD and despite full-donor chimerism are bolded and italicized. ATG: anti-thymoglobulin; Alem: alemtuzumab; BAL: bronchoalveolar lavage; Bu: busulfan; Cy: cyclophosphamide; Flu: fludarabine; GVHD: graft-versus-host disease; MAC: myeloablative conditioning regimen; Mel: melphalan; MMRD: mismatched-related donor; MSD: matched sibling donor; NA: not applicable; NR: not reported; NP: not performed; RIC: reduced-intensity conditioning regimen; Rx: rituximab; Treo: treosulfan; TMA: thrombotic microangiopathy; TPN: total parenteral nutrition; TREC: T cell receptor excision circles; URD: unrelated donor.

a Microbiological, endocrine, metabolic, and immunological data for digestive tract involvement were negative during symptoms: viral, parasitic, and bacterial stool investigation; anti-AIE75, -*Saccharomyces*, transglutaminase antibodies; amino acids chromatography, congenital glycosylation disorder testing; thyroid tests.

b Digestive tract histology showing total villous atrophy with inflammation at 7 mo and villous regeneration with B and T cell infiltrates at 12 mo.

c CD34-positive selection of the graft. Busulfan with measured total AUC 68,940 ng/mLxh, fludarabine 160 mg/m², and thymoglobulin 7.5 mg/kg.

Many patients developed unusually severe and even fatal endothelial disease following allo-HSCT, exceeding the severity expected according to intensity of CR (Table 2).

All transplanted patients died, except one who had revertant mosaicism in both hematopoietic and non-hematopoietic tissues (5). Post-transplant organ complications worsened or persisted long after HSCT despite full-donor chimerism, suggesting a likely nonimmune contribution to the severe phenotype, particularly endothelial dysfunction.

Both dominant-negative PSMB10 and dominant-negative PSMB9 diseases (4) share a common pathogenic mechanism through disruption of PSMB9 stability and immunoproteasome assembly. The corresponding *Psmb9* mouse model also had strong similarities to the Gly170Trp/Gly170Trp *Psmb10* with immunoproteasome assembly impairment (12). Yet, both diseases have distinct clinical trajectories. Both conditions result in profound T and B cell lymphopenia, but severe infections are reported for *PSBM10* variants only. Endothelial perturbations seem to occur in both, with VOD for *PSMB10* variants and very early liver cirrhosis with fatal liver failure and severe pulmonary hypertension for *PSMB9* variants. More strikingly, while *PSMB9* variants as well as autosomal recessive PRAAS patients exhibit systemic inflammation with elevated CRP levels, elevated type-1 interferon signature, and occasional central nervous system inflammation, these features were not prominent in all reported patients harboring deleterious heterozygous *PSMB10* variants except during infection or VOD flare, with normal type-1 interferon signature in the both tested patients. Further comparisons between proteasome-associated diseases are provided in Table 3.

**Table 3.** Comparative clinical features of dominant-negative PSMB10 disease, dominant-negative PSMB9 disease, POMP deficiency (PRAID), and autosomal recessive PRAAS

​	Dominant-negative *PSMB10* (10 patients)	Dominant-negative *PSMB9* (2 patients)	POMP deficiency (5 patients)	Autosomal recessive PRAAS (<100 patients)
Inheritance	De novo, heterozygous	De novo, heterozygous	De novo, heterozygous	Autosomal recessive
Age at onset	Neonatal/infancy	Neonatal/infancy	Infancy	Infancy/early childhood
Systemic inflammation	− (not prominent)	++	++	++
Liver involvement	+++ (frequent, severe VOD, liver failure, and high mortality)	++ (early cirrhosis, CMV-triggered hepatitis, and fatal liver failure)	+ (mild-moderate, rarely severe)	+ (mild-moderate, hepatomegaly common, failure rare)
Gastrointestinal involvement	++ (mild to severe enteropathy)	​	​	​
Pulmonary hypertension	−	++	−	−
Skin involvement	Erythroderma and severe atopic dermatitis	Erythroderma and neonatal rash	Neutrophilic dermatosis	Panniculitis, lipodystrophy and chronic rash
Lipodystrophy	−	−	−	++
Muscle/Joint involvement	−	Myositis	−	Myositis, muscle atrophy, and joint contractures
Infections	++, recurrent opportunistic infections	−	++, recurrent opportunistic infections	+/−
Immunological Features	Severe SCID/CID	T cell lymphopenia and CD8^+^ T cell loss	CID	Mild to moderate lymphopenia
​	Profound T and B cell lymphopenia	​	T and B cell lymphopenia	Pancytopenia (variable)
​	Low naïve T cell (variable)	​	​	Hypergammaglobulinemia
​	Hypogammaglobulinemia	​	​	​
IFN-I signature	Normal in 2 tested patients	High	High in 1	High
​	​	​	Normal in 1 (under treatment)	​
JAK-inhibitor	Not tested	Efficient on pulmonary hypertension and autoinflammation (1 patient)	Might control autoinflammation increased risk of infection	Indicated for autoinflammation
HSCT outcome	Very high morbi-mortality	1 patient transplanted and cured	2 patients transplanted and cured	1 PSMB4-deficient patient and cured

HLH: hemophagocytic lymphohistiocytosis; PRAID: POMP-related autoinflammation and immune dysregulation.

Immune parameters showed mostly a severe and global lymphocyte defect, but one out of the six p.Gly201Arg patients had normal naive T cells and repertoire at onset, while one of the two p.Ser208Phe patients (PIII) had almost normal immune parameters. Radiation sensitivity was normal in two, but strongly decreased in one (no other genetic variant relevant for DNA repair by WES). The explanation of these discrepancies is lacking, as is the mechanism of *Pneumocystis* pneumonia in PIII with normal immune parameters.

While a thymic defect was suggested (6), this fails to explain the B cell development defect and the likely extra-hematopoietic gut and liver involvements. Impaired lymphocyte survival may explain the global immune defect, especially under stress (12), and defective thymocyte survival may underlie the abnormal PROMIDISα signature during TCRα rearrangement (10), as previously described in both human and mice (17, 18). Interestingly, the severe lymphopenia in the TUB6 mouse, which carries the p.Gly170Trp *Psmb10* variant, is caused by impaired lymphocyte survival secondary to immunoproteasome assembly (12). The impairment of naïve CD4 T cells and B cell also argue against the proteasome-mediated peptide presentation through HLA class I molecules, which would only impact CD8 T cells, which is further evidenced by preserved HLA-I-peptide presentation in the dominant-negative *Psmb9* mouse model (4). Finally, impaired proliferation or survival of *Psmb9*, *Psmb10*, or *Psmb10/Psmb8* double-knockout CD8 T cells after adoptive transfer in wild-type-infected mice is suggestive of an intrinsic T cell defect occurring after T cell development in the thymus (19, 20, 21).

The p.Asp205Ala and p.Ser208Phe *PSMB10* variants exerted a dominant-negative effect on proteasome assembly. Treise et al. corroborated the dominant-negative effect in a mouse model, with a much more severe phenotype in Gly170Trp/WT *Psmb10* mice compared to Gly170Trp/Gly170Trp *Psmb10* mice, and a stronger T cell defect in Gly170Trp/WT *Psmb10* than in *Psmb10* −/− mice on the C3HeB/FeJ background (12). Indeed, the complete absence of PSMB8, PSMB9, PMSB10, or both PSBM8 and PSMB10 in mice results in a very mild immune phenotype, with increased susceptibility to infection and normal T cell development, the missing subunit being replaced by others and providing relatively effective proteasome functions (19, 20, 21, 22, 23). The dominant-negative effect was particularly marked with reduced steady-state levels of the PSMB9, as shown in PIII-derived T cells (Fig. 3 B) and consistent with our in vitro findings showing that the p.Ser208Phe *PSMB10* variant exerted a dominant-negative effect on endogenous PSMB9 expression (Fig. 2 C). It is worth noting that the effect in patient-derived T cells was less pronounced compared to the HEK293T-based overexpression system. This discrepancy likely reflects physiological differences between the two systems. In contrast to HEK293T cells, T cells exhibit constitutive immunoproteasome assembly, which may partially buffer or compensate for the dominant-negative effect of the p.Ser208Phe *PSMB10* variant. Besides, because HEK293T cells express only low levels of immunoproteasomes, the downregulation of endogenous PSMB9 in this context did not result in a detectable accumulation of ubiquitinated proteins (Fig. S2).

This phenotype closely resembles that reported for the p.Gly201Arg variant, which was similarly associated with diminished levels of mature PSMB9 in HeLa cells (6). Notably, neither variant was associated with accumulation of the PSMB9 precursor subunit, suggesting that the variants affected PSMB9 stability or expression rather than its processing. This observation implies that PSMB9 and PSMB10 precursor subunits may interact early during immunoproteasome assembly, with the dysfunction of one subunit leading to the destabilization and degradation of the other. This model aligns with current understanding of immunoproteasome biogenesis, wherein PSMB8 and PSMB9 are believed to bind cooperatively to the α-ring to form half-proteasomes at an early stage of assembly (24).

As shown in Fig. 3 B, SDS-PAGE and western blot analysis revealed decreased levels of the PSMB10 mature subunit in PIII-derived T cells. This finding aligns with our in vitro results, where the V5/HIS-tagged p.Ser208Phe PSMB10 construct exhibited reduced expression of both the PSMB10 precursor and mature subunits (Fig. 2 B). However, the absence of detectable PSMB10 precursor in patient T cells leaves it unclear whether the reduced levels of the mature subunit are due to diminished steady-state expression or impaired precursor processing. Unfortunately, we could not directly evaluate PSMB10 incorporation into proteasomes in patient cells, as the PSMB10-specific antibody was unsuitable for native-PAGE and western blot analysis.

Furthermore, the association of 26S proteasomes with PA28 appears to be influenced by PSMB10, as levels of hybrid (i.e., 19S-20S-PA28) complexes were reduced in patient T cells (Fig. 3 A). While previous studies suggest that PA28 is essential for immunoproteasome assembly (25), whether PA28 preferentially binds proteasomes containing PSMB10 remains unclear. It is tempting to speculate that PSMB10 incorporation induces structural changes that enhance PA28 recruitment, but this possibility warrants further exploration.

Finally, stress conditions, such as infection for PII and PIII, or alkylating antineoplastic agents for PI, induce a switch from the constitutive proteasome to immunoproteasome in the liver (21, 26). Thus, in patients with this particular immunoproteasome deficiency, hepatocytes would depend solely on a defective proteasome, strongly impairing cellular homeostasis (27). This mechanism might apply to other tissues, notably the digestive tract or endothelial cells.

HSCT was successful in a single patient with a heterozygous *PSMB9* variant (28) despite potential vascular complications (pulmonary hypertension) and a seemingly similar pathophysiology. However, the pathophysiology may differ significantly, and contrary to dominant-negative PSMB10 disease, patients with heterozygous *PSMB9* variants present with prominent systemic autoinflammation and elevated type-I interferon signature that responded to JAK inhibitors in the transplanted patient (28).

Additionally, the contrasting HSCT outcomes in proteasome disorders could reflect differential tissue resilience of proteasome assembly. In POMP deficiency and autosomal recessive PRAAS, such as biallelic *PSMB4* variants (29), a relatively preserved proteasome assembly in non-hematopoietic tissues under stress condition might allow these organs to tolerate HSCT conditioning stress and maintain homeostasis after transplant. Conversely, dominant-negative *PSMB10* variants may cause a more severely impaired proteasome assembly across several tissues in case of stress, rendering hepatocytes, endothelial cells, and digestive tract unable to sustain the additional stress of HSCT conditioning.

In conclusion, we identified novel heterozygous monoallelic variants in the PSMB10 immunoproteasome subunit gene in three patients with CID or SCID. These variants likely impair lymphocyte development and survival and highlight an association to severe liver and endothelial dysfunction. PSMB10-deficient patients should not undergo HSCT without careful consideration of potential liver and endothelial toxicities.

## Materials and methods

### Cell culture

HEK293T cells (ATCC) were cultured in Dulbecco’s modified Eagle’s medium supplemented with 10% fetal bovine serum and 1% penicillin/streptomycin (all from Life Technologies). Peripheral blood mononuclear cells (PBMCs) were isolated from PII using Biocoll-based density gradient centrifugation after informed consent. For T cell expansion, PBMCs were co-cultured with irradiated allogeneic PBMCs as feeder cells in the presence of 150 U/ml IL-2 and 1 µg/ml phytohemagglutinin (PHA)-L, following the protocol described by Fonteneau et al. (30). Age-matched PBMC-derived T cells from healthy individuals served as controls. These samples were obtained from the BioTND-UPS biobank, which has been approved by the Ethics Committee for Personal Data Protection (CCP-Ouest IV, file #06/15, reference MESR DC-2017-2987).

### Plasmid construction and transfection

The cDNA encoding the full-length PSMB10 protein subunit (also known as β2i or MECL-1) was amplified by RT-PCR from total RNA extracted from T cells and cloned into the pcDNA3.1/V5-His-TOPO expression vector (Invitrogen) to generate a C-terminally V5/HIS-tagged PSMB10 construct. Site-directed mutagenesis was performed to introduce single mutations into the PSMB10-V5/HIS construct, resulting in the generation of p.(D205A), p.(S208F), p.(G209W), p.(F14S), and p.(F14del) PSMB10 variants. Mammalian cell transfections were carried out using the jetPRIME transfection reagent (Polyplus-Sartorius) following the manufacturer’s instructions.

### Semiquantitative RT-PCR analysis

Total RNA was extracted from HEK293T cells using the NucleoSpin RNA Kit (Macherey-Nagel) according to the manufacturer’s instructions. For cDNA synthesis, 1,000 ng of total RNA was reverse transcribed using ProtoScript II Reverse transcriptase (New England Biolabs, Inc.). PCR amplification was performed using a reaction mixture containing 20 ng cDNA, 10 µM each of forward (PSMB10) and reverse (BGH polyadenylation signal) primers, and 1× PrimeSTAR MAX DNA polymerase Premix (Takara) in a final volume of 25 μl. Expression of glyceraldehyde-3-phosphate dehydrogenase (GAPDH) mRNA was used as a housekeeping control. PCR products were separated by electrophoresis on a 1.5% agarose gel in Tris-borate-EDTA (TBE) buffer, stained with ethidium bromide, and visualized under UV light using a Bio-Rad ChemiDoc Imaging System.

### Native-PAGE, SDS-PAGE, and western blotting

At 24 h after transfection, HEK293T cells were harvested, washed twice with PBS, and resuspended in Tris-DTT-Glycerol (TSDG) lysis buffer containing 10 mM Tris (pH 7.5), 10 mM NaCl, 25 mM KCl, 1 mM MgCl_2_, 0.1 mM ethylenediaminetetraacetic acid, 2 mM dithiothreitol, 2 mM ATP, and 10% (vol/vol) glycerol. Non-denaturing protein extraction was performed by subjecting the samples to five freeze-thaw cycles using liquid nitrogen and water. Protein concentration was determined using a standard Bradford assay. For non-denaturing separation, 20 µg of protein lysates were mixed with Native PAGE Sample Buffer (Thermo Fisher Scientific), loaded onto 3–12% precast native-PAGE gels (Thermo Fisher Scientific), and resolved by electrophoresis at 150 V for 1 h, followed by 250 V for 30 min. Proteasome bands were visualized by incubating the gels with 0.1 mM Suc–Leu–Leu–Val–Tyr–AMC (Bachem) fluorogenic substrate for 20 min at 37°C in 20 mM Tris, 5 mM MgCl_2_, and 2 mM ATP. Fluorescence was detected and quantified using a ChemiDoc MP Imaging System (Bio-Rad). For SDS-PAGE, 10–20 µg of protein lysates were mixed with NuPAGE LDS Sample Buffer (Thermo Fisher Scientific), heated at 95°C for 5 min, and separated under denaturing conditions on 4–12% precast NuPAGE gels (Thermo Fisher Scientific). For western blotting, protein lysates separated by either native- or SDS-PAGE were transferred onto polyvinylidene difluoride (PVDF) membranes, blocked with 3% BSA, and incubated overnight with primary antibodies specific to V5 (CloneSV5-Pk1; Thermo Fisher Scientific), PSMA1 (clone MC20; Enzo Life Sciences Inc.), PSME1 (clone D1C10; Cell Signaling Technology), UBE2L6 (clone 2F12-1F4, antibodies online), PSMB8 (clone A12, Santa Cruz Biotechnology Inc.), PSMB9 (clone E7J1L; Cell Signaling Technology), PSMB10 (clone E6R7O Cell Signaling Technology), K48-linked ubiquitin-modified proteins (clone D9D5; Cell Signaling Technology), β-actin (clone C4; Santa Cruz Biotechnology, Inc.), and GAPDH (clone 14C10; Cell Signaling Technology). Peroxidase-conjugated secondary antibodies (anti-rabbit and anti-mouse) were used at a 1:5,000 dilution. Signal detection was performed using enhanced chemiluminescence and visualized with a ChemiDoc MP Imaging System (Bio-Rad). Densitometric analysis was carried out by quantifying the intensity of immunoreactive signals using freely available Fiji image processing program (https://imagej.net/software/fiji/downloads).

### PROMIDISα

The PROMIDISα signature was determined from whole PBMCs as previously described with slight modifications (10). Briefly, RNA was purified from PBMC and reverse transcribed. A subset of TCRα repertoire was then amplified by multiplex PCR using eight forward Vα primers representing proximal (TRAV35 5′-CTC​AGT​TTG​GTA​TAA​CCA​GAA​AGG​A-3′ and TRAV41 5′-GAT​TAA​TTG​CCA​CAA​TAA​ACA​TAC​AGG-3′), middle (TRAV20 5′-GCC​ACA​TTA​ACA​AAG​AAG​GAA​AGC​T-3′, TRAV21 5′-GCC​TCG​CTG​GAT​AAA​TCA​TCA​GGA-3′, and TRAV23 5′-CAC​AAT​CTC​CTT​CAA​TAA​AAG​TGC​CA-3′), and distal (TRAV1 5′-AGG​TCG​TTT​TTC​TTC​ATT​CCT​TAG​TC-3′, TRAV5 5′-AAA​CAA​GAC​CAA​AGA​CTC​ACT​GTT​C-3′, and TRAV10 5′-TAC​AGC​AAC​TCT​GGA​TGC​AGA​CAC-3′) Vα segments and a constant region (Cα 5′-GCAGGGTCAGGGTTCTGGATAT-3′)-specific reverse primer. PCR products were further processed for NGS Illumina sequencing. TCR-Vα and Jα usage was assigned through IMGT/HighV-Quest (https://www.imgt.org/HighV-QUEST/home.action) (31) and quantified using custom R script. PROMIDISα signature was evaluated through hierarchical clustering using FactoMineR (32) against in-house PROMIDISα database containing signatures for 24 healthy controls, 18 patients with various defects in V(D)J recombination and/or DNA repair, and 35 ataxia telangiectasia patients with ATM gene mutations.

### Cellular sensitivity to ionizing radiation

PBMCs isolated from heparinized blood were subjected to increasing doses of ionizing radiations. After resting 4 h in complete culture medium (RPMI 10% FCS) to allow for DNA repair, T lymphocytes were activated with CD3/CD28-coated beads (Dynabeads) and cultured in the presence of IL2 (10^3^ µ/ml) in 48-well plates. After 6 days of culture, cells were recovered, washed, and counted through flow cytometry. The survival fraction represents the cell count of irradiated cells relative to untreated cells. Data were compared to the mean survival of PBMCs obtained from 15 healthy controls and 31 radiosensitive controls (4 patients with Lig4 syndrome with mutations in DNA ligase IV and 27 ATM-mutated ataxia telangiectasia patients).

### Statistics

Pairwise comparisons from data generated by western blotting were typically presented as means ± SEM and analyzed by paired *t* test. All charts and statistical analyses were generated using GraphPad Prism version 8. A P value <0.05 was considered significant.

### Online supplemental material

Fig. S1 shows semiquantitative RT-PCR analysis of PSMB10 mRNA expression in HEK293T cells transfected with wild-type and mutant PSMB10-V5/HIS constructs. Fig. S2 shows western blot analysis of K48-linked polyubiquitinated proteins in HEK293T cells expressing PSMB10 variants following IFN-γ stimulation (200 U/ml).

## Supplementary Material

SourceData F2 is the source file for Fig. 2.

SourceData F3 is the source file for Fig. 3.

SourceData FS1 is the source file for Fig. S1.

SourceData FS2 is the source file for Fig. S2.

## Data Availability

The data underlying Figs. 1, 2, 3, S1, and S2 and tables are available in the published article and its online supplemental material.

## References

[1] Zhang, J., P. Tao, N.T. Deuitch, X. Yu, I. Askentijevich, and Q. Zhou. 2024. Proteasome-associated syndromes: Updates on genetics, clinical manifestations, pathogenesis, and treatment. J. Clin. Immunol. 44:88. 10.1007/s10875-024-01692-y 38578475

[2] Livneh, I., V. Cohen-Kaplan, C. Cohen-Rosenzweig, N. Avni, and A. Ciechanover. 2016. The life cycle of the 26S proteasome: From birth, through regulation and function, and onto its death. Cell Res. 26:869–885. 10.1038/cr.2016.86 27444871 PMC4973335

[3] Aksentijevich, I., and O. Schnappauf. 2021. Molecular mechanisms of phenotypic variability in monogenic autoinflammatory diseases. Nat. Rev. Rheumatol. 17:405–425. 10.1038/s41584-021-00614-1 34035534

[4] Kanazawa, N., H. Hemmi, N. Kinjo, H. Ohnishi, J. Hamazaki, H. Mishima, A. Kinoshita, T. Mizushima, S. Hamada, K. Hamada, . 2021. Heterozygous missense variant of the proteasome subunit β-type 9 causes neonatal-onset autoinflammation and immunodeficiency. Nat. Commun. 12:6819. 10.1038/s41467-021-27085-y 34819510 PMC8613290

[5] van der Made, C.I., S. Kersten, O. Chorin, K.R. Engelhardt, G. Ramakrishnan, H. Griffin, I. Schim van der Loeff, H. Venselaar, A.R. Rothschild, M. Segev, . 2024. Expanding the PRAAS spectrum: *De novo* mutations of immunoproteasome subunit β-type 10 in six infants with SCID-Omenn syndrome. Am. J. Hum. Genet. 111:791–804. 10.1016/j.ajhg.2024.02.013 38503300 PMC11023912

[6] Kuehn, H.S., M. Bosticardo, A.C. Arrieta, J.L. Stoddard, F. Pala, J.E. Niemela, A.A. Gil Silva, P.L. King, A. Esteve-Sole, A. Naveen, . 2025. Thymic and T-cell intrinsic critical roles associated with Severe Combined Immunodeficiency and Omenn syndrome due to a heterozygous variant (G201R) in PSMB10. J. Allergy Clin. Immunol. 155:1378–1385.e2. 10.1016/j.jaci.2024.12.1082 39734035 PMC11972880

[7] Lankester, A.C., M.H. Albert, C. Booth, A.R. Gennery, T. Güngör, M. Hönig, E.C. Morris, D. Moshous, B. Neven, A. Schulz, . 2021. EBMT/ESID inborn errors working party guidelines for hematopoietic stem cell transplantation for inborn errors of immunity. Bone Marrow Transpl. 56:2052–2062. 10.1038/s41409-021-01378-8 PMC8410590 34226669

[8] Santos, R.L.A., L. Bai, P.K. Singh, N. Murakami, H. Fan, W. Zhan, Y. Zhu, X. Jiang, K. Zhang, J.P. Assker, . 2017. Structure of human immunoproteasome with a reversible and noncompetitive inhibitor that selectively inhibits activated lymphocytes. Nat. Commun. 8:1692. 10.1038/s41467-017-01760-5 29167449 PMC5700161

[9] Fournier, B., N. Mahlaoui, D. Moshous, and J.P. de Villartay. 2022. Inborn errors of immunity caused by defects in the DNA damage response pathways: Importance of minimizing treatment-related genotoxicity. Pediatr. Allergy Immunol. 33:e13820. 10.1111/pai.13820 35754136 PMC9327728

[10] Berland, A., J. Rosain, S. Kaltenbach, V. Allain, N. Mahlaoui, I. Melki, A. Fievet, C. Dubois d’Enghien, M. Ouachée-Chardin, L. Perrin, . 2019. PROMIDISα: A T-cell receptor α signature associated with immunodeficiencies caused by V(D)J recombination defects. J. Allergy Clin. Immunol. 143:325–334.e2. 10.1016/j.jaci.2018.05.028 29906526

[11] Cheng, J., G. Novati, J. Pan, C. Bycroft, A. Žemgulytė, T. Applebaum, A. Pritzel, L.H. Wong, M. Zielinski, T. Sargeant, . 2023. Accurate proteome-wide missense variant effect prediction with AlphaMissense. Science. 381:eadg7492. 10.1126/science.adg7492 37733863

[12] Treise, I., E.M. Huber, T. Klein-Rodewald, W. Heinemeyer, S.A. Grassmann, M. Basler, T. Adler, B. Rathkolb, L. Helming, C. Andres, . 2018. Defective immuno- and thymoproteasome assembly causes severe immunodeficiency. Sci. Rep. 8:5975. 10.1038/s41598-018-24199-0 29654304 PMC5899138

[13] Groettrup, M., S. Standera, R. Stohwasser, and P.M. Kloetzel. 1997. The subunits MECL-1 and LMP2 are mutually required for incorporation into the 20S proteasome. Proc. Natl. Acad. Sci. USA. 94:8970–8975. 10.1073/pnas.94.17.8970 9256419 PMC22989

[14] Guillaume, B., J. Chapiro, V. Stroobant, D. Colau, B. Van Holle, G. Parvizi, M.-P. Bousquet-Dubouch, I. Théate, N. Parmentier, and B.J. Van den Eynde. 2010. Two abundant proteasome subtypes that uniquely process some antigens presented by HLA class I molecules. Proc. Natl. Acad. Sci. USA. 107:18599–18604. 10.1073/pnas.1009778107 20937868 PMC2972972

[15] Papendorf, J.J., F. Ebstein, S. Alehashemi, D.G.P. Piotto, A. Kozlova, M.T. Terreri, A. Shcherbina, A. Rastegar, M. Rodrigues, R. Pereira, . 2023. Identification of eight novel proteasome variants in five unrelated cases of proteasome-associated autoinflammatory syndromes (PRAAS). Front. Immunol. 14:1190104. 10.3389/fimmu.2023.1190104 37600812 PMC10436547

[16] Sarrabay, G., D. Méchin, A. Salhi, G. Boursier, C. Rittore, Y. Crow, G. Rice, T.-A. Tran, R. Cezar, D. Duffy, . 2020. PSMB10, the last immunoproteasome gene missing for PRAAS. J. Allergy Clin. Immunol. 145:1015–1017.e6. 10.1016/j.jaci.2019.11.024 31783057

[17] Okada, S., J.G. Markle, E.K. Deenick, F. Mele, D. Averbuch, M. Lagos, M. Alzahrani, S. Al-Muhsen, R. Halwani, C.S. Ma, . 2015. IMMUNODEFICIENCIES. Impairment of immunity to Candida and Mycobacterium in humans with bi-allelic RORC mutations. Science. 349:606–613. 10.1126/science.aaa4282 26160376 PMC4668938

[18] Guo, J., A. Hawwari, H. Li, Z. Sun, S.K. Mahanta, D.R. Littman, M.S. Krangel, and Y.-W. He. 2002 May. Regulation of the TCRalpha repertoire by the survival window of CD4(+)CD8(+) thymocytes. Nat. Immunol. 3:469–476. 10.1038/ni791 11967541

[19] Basler, M., J. Moebius, L. Elenich, M. Groettrup, and J.J. Monaco. 2006. An altered T cell repertoire in MECL-1-deficient mice. J. Immunol. 176:6665–6672. 10.4049/jimmunol.176.11.6665 16709825

[20] Basler, M., U. Beck, C.J. Kirk, and M. Groettrup. 2011. The antiviral immune response in mice devoid of immunoproteasome activity. J. Immunol. 187:5548–5557. 10.4049/jimmunol.1101064 22013127

[21] Van Kaer, L., P.G. Ashton-Rickardt, M. Eichelberger, M. Gaczynska, K. Nagashima, K.L. Rock, A.L. Goldberg, P.C. Doherty, and S. Tonegawa. 1994. Altered peptidase and viral-specific T cell response in LMP2 mutant mice. Immunity. 1:533–541. 10.1016/1074-7613(94)90043-4 7600282

[22] Zaiss, D.M.W., N. de Graaf, and A.J. Sijts. 2008. The proteasome immunosubunit multicatalytic endopeptidase complex-like 1 is a T-cell-intrinsic factor influencing homeostatic expansion. Infect. Immun. 76:1207–1213. 10.1128/IAI.01134-07 18160473 PMC2258853

[23] Tu, L., C. Moriya, T. Imai, H. Ishida, K. Tetsutani, X. Duan, S. Murata, K. Tanaka, C. Shimokawa, H. Hisaeda, and K. Himeno. 2009. Critical role for the immunoproteasome subunit LMP7 in the resistance of mice to Toxoplasma gondii infection. Eur. J. Immunol. 39:3385–3394. 10.1002/eji.200839117 19830724

[24] Watanabe, A., H. Yashiroda, S. Ishihara, M. Lo, and S. Murata. 2022. The molecular mechanisms governing the assembly of the immuno- and thymoproteasomes in the presence of constitutive proteasomes. Cells. 11:1580. 10.3390/cells11091580 35563886 PMC9105311

[25] Preckel, T., W.P. Fung-Leung, Z. Cai, A. Vitiello, L. Salter-Cid, O. Winqvist, T.G. Wolfe, M. Von Herrath, A. Angulo, P. Ghazal, . 1999. Impaired immunoproteasome assembly and immune responses in PA28−/− mice. Science. 286:2162–2165. 10.1126/science.286.5447.2162 10591649

[26] Khan, S., M. van den Broek, K. Schwarz, R. de Giuli, P.A. Diener, and M. Groettrup. 2001. Immunoproteasomes largely replace constitutive proteasomes during an antiviral and antibacterial immune response in the liver. J. Immunol. 167:6859–6868. 10.4049/jimmunol.167.12.6859 11739503

[27] Seifert, U., L.P. Bialy, F. Ebstein, D. Bech-Otschir, A. Voigt, F. Schröter, T. Prozorovski, N. Lange, J. Steffen, M. Rieger, . 2010. Immunoproteasomes preserve protein homeostasis upon interferon-induced oxidative stress. Cell. 142:613–624. 10.1016/j.cell.2010.07.036 20723761

[28] Kataoka, S., N. Kawashima, Y. Okuno, H. Muramatsu, S. Miwata, K. Narita, M. Hamada, N. Murakami, R. Taniguchi, D. Ichikawa, . 2021. Successful treatment of a novel type I interferonopathy due to a de novo PSMB9 gene mutation with a Janus kinase inhibitor. J. Allergy Clin. Immunol. 148:639–644. 10.1016/j.jaci.2021.03.010 33727065

[29] Verhoeven, D., D. Schonenberg-Meinema, F. Ebstein, J.J. Papendorf, P.A. Baars, E.M.M. van Leeuwen, M.H. Jansen, A.C. Lankester, M. van der Burg, S. Florquin, . 2022. Hematopoietic stem cell transplantation in a patient with proteasome-associated autoinflammatory syndrome (PRAAS). J. Allergy Clin. Immunol. 149:1120–1127.e8. 10.1016/j.jaci.2021.07.039 34416217

[30] Fonteneau, J.F., M. Larsson, S. Somersan, C. Sanders, C. Münz, W.W. Kwok, N. Bhardwaj, and F. Jotereau. 2001. Generation of high quantities of viral and tumor-specific human CD4+ and CD8+ T-cell clones using peptide pulsed mature dendritic cells. J. Immunol. Methods. 258(1–2):111–126. 10.1016/s0022-1759(01)00477-x 11684128

[31] Li, S., M.P. Lefranc, J.J. Miles, E. Alamyar, V. Giudicelli, P. Duroux, J.D. Freeman, V.D.A. Corbin, J.-P. Scheerlinck, M.A. Frohman, . 2013. IMGT/HighV QUEST paradigm for T cell receptor IMGT clonotype diversity and next generation repertoire immunoprofiling. Nat. Commun. 4:2333. 10.1038/ncomms3333 23995877 PMC3778833

[32] Lê, S., J. Josse, and F. Husson. 2008. FactoMineR: An *R* package for multivariate analysis. J. Stat. Soft. 25. 10.18637/jss.v025.i01

