# Supplementary figures and images for: Novel de novo dominant PSMB10 variants in three patients with immune deficiency and liver disease
Source: J Hum Immun. 2025 Nov 18;2(1):e20250096. doi: 10.70962/jhi.20250096 (PMC13177760; doi:10.70962/jhi.20250096)

Fig. 2A

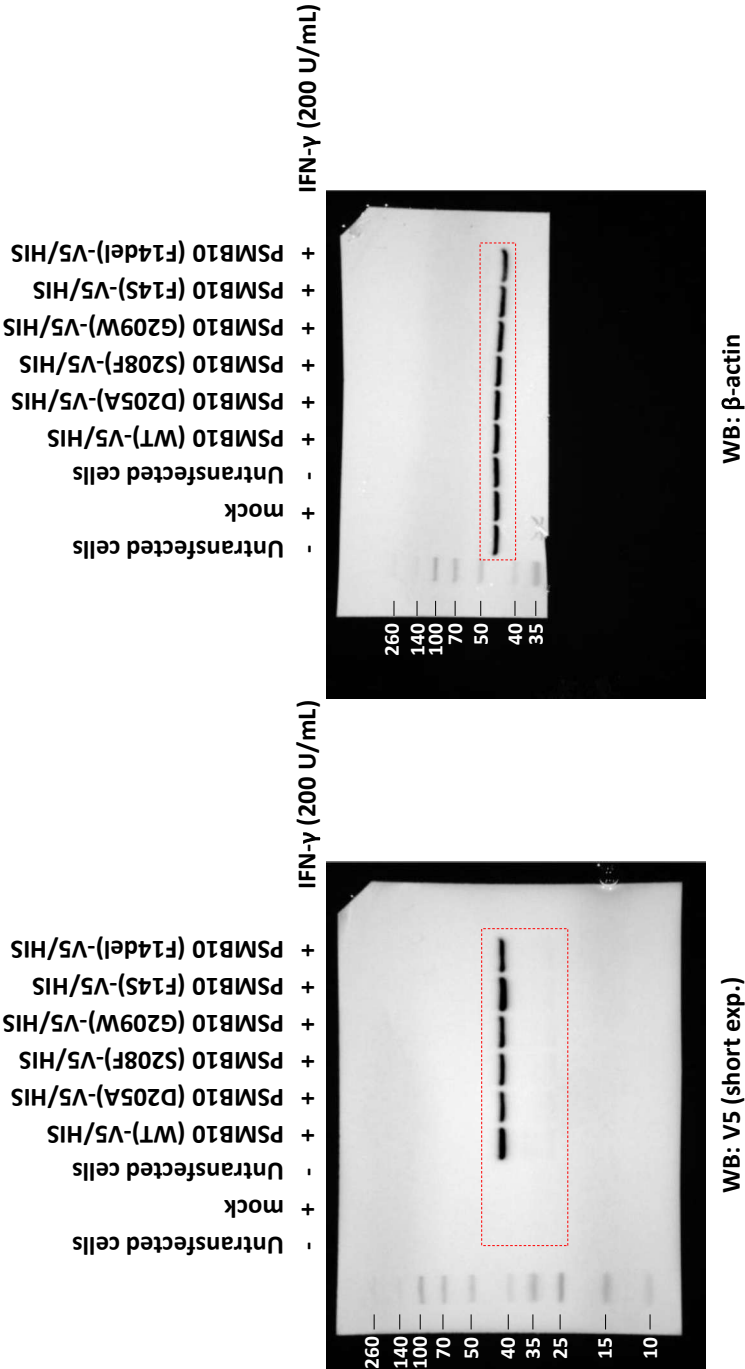

Fig. 2A

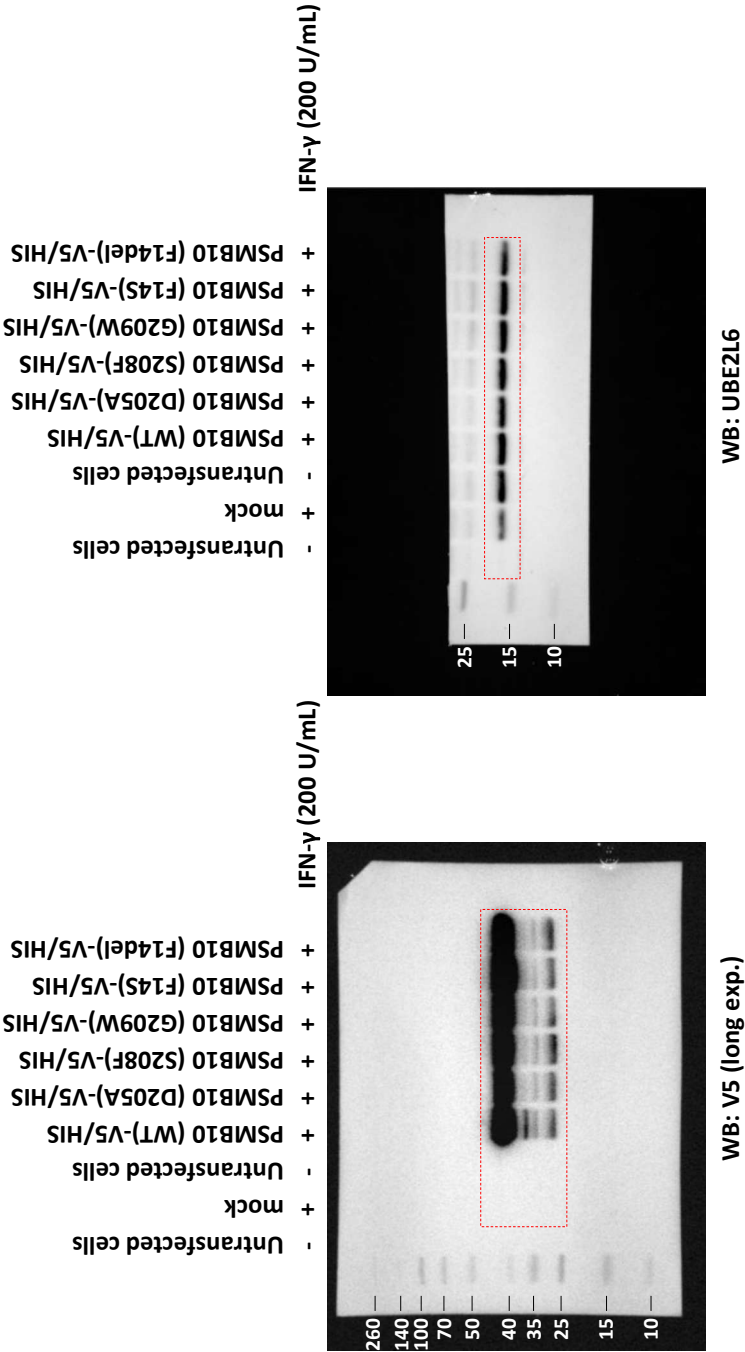

**Fig. 2B**

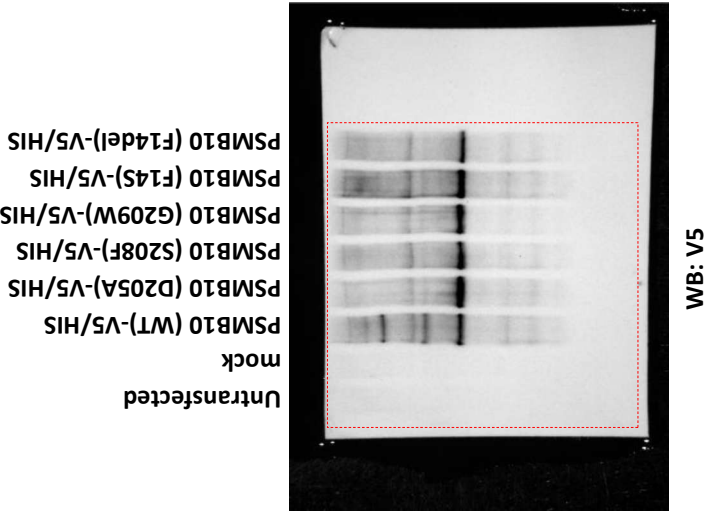

Fig. 2C

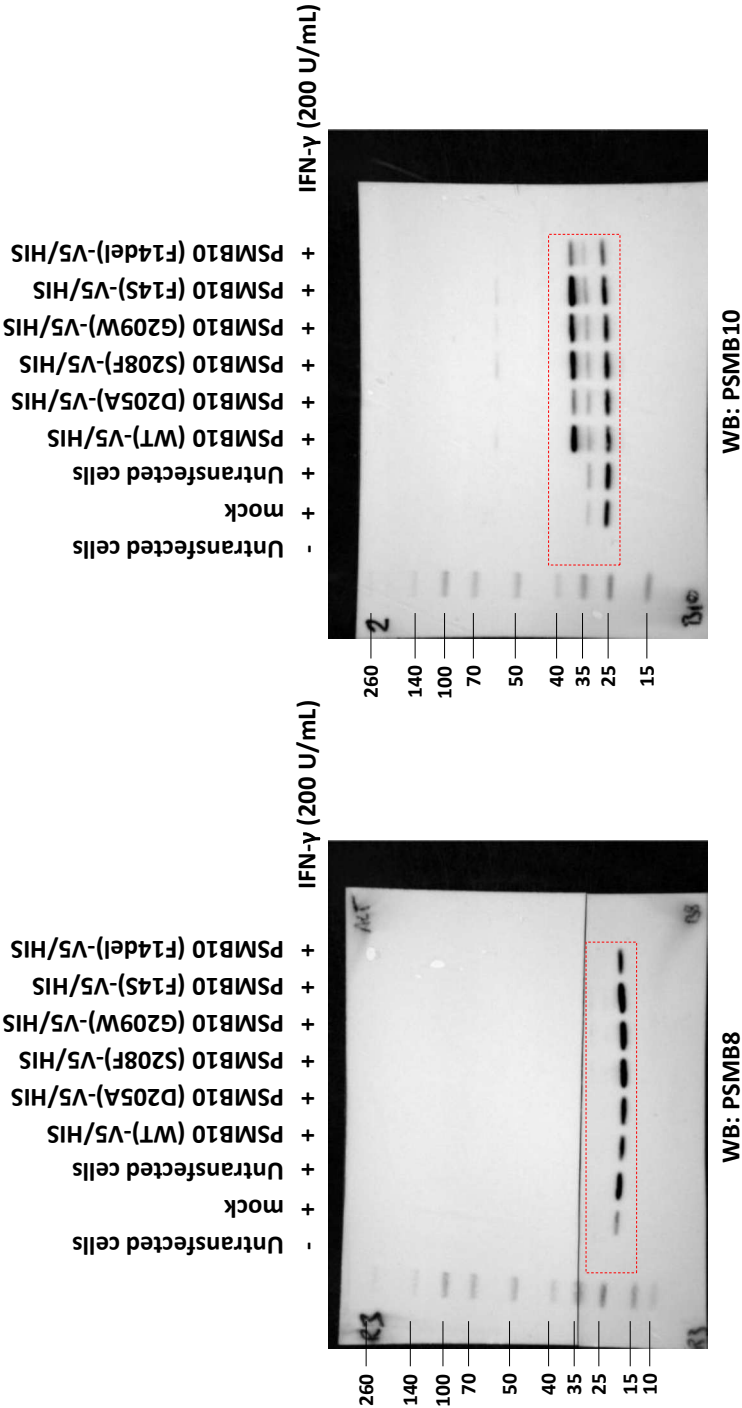

Fig. 2C

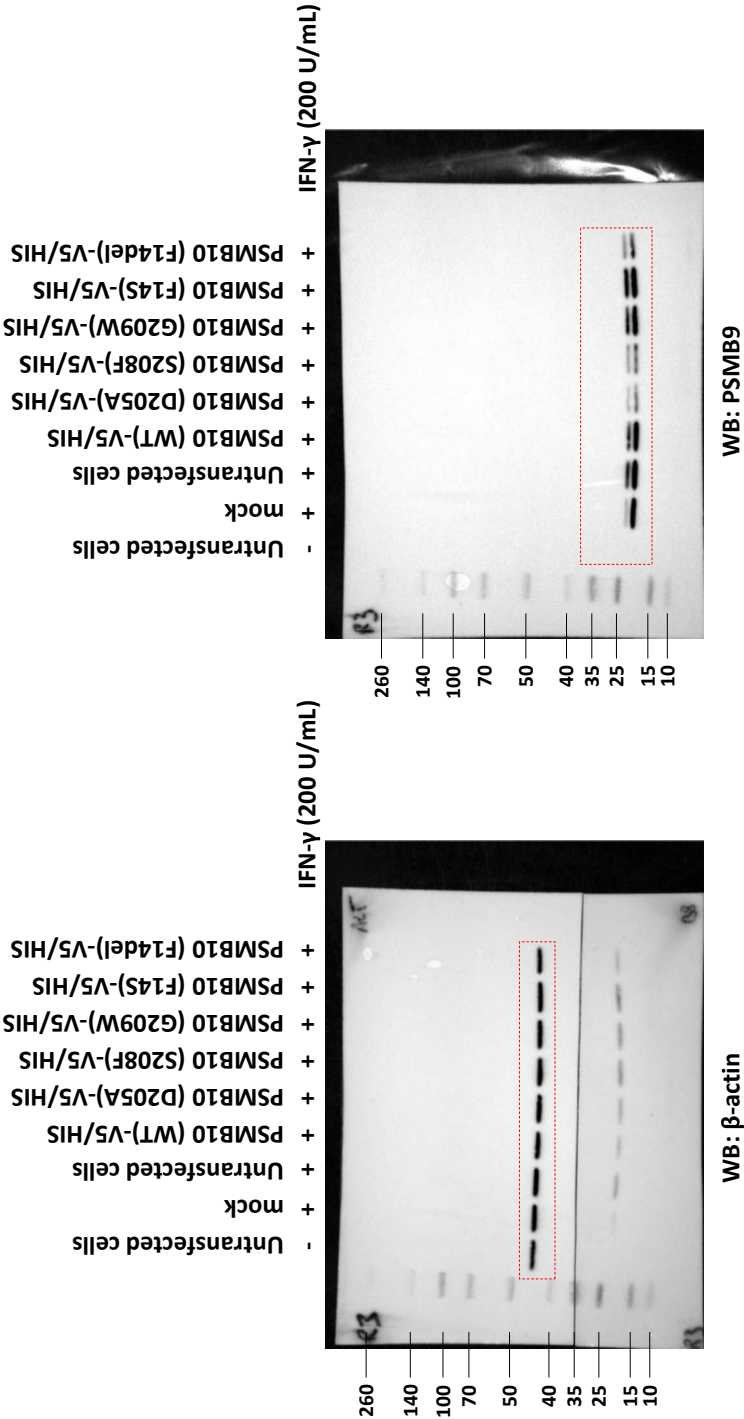

Supplement: SourceData F2 — is the source file for Fig. 2. [file jhi_20250096_sourcedataf2.pdf]

**Fig. 3A**

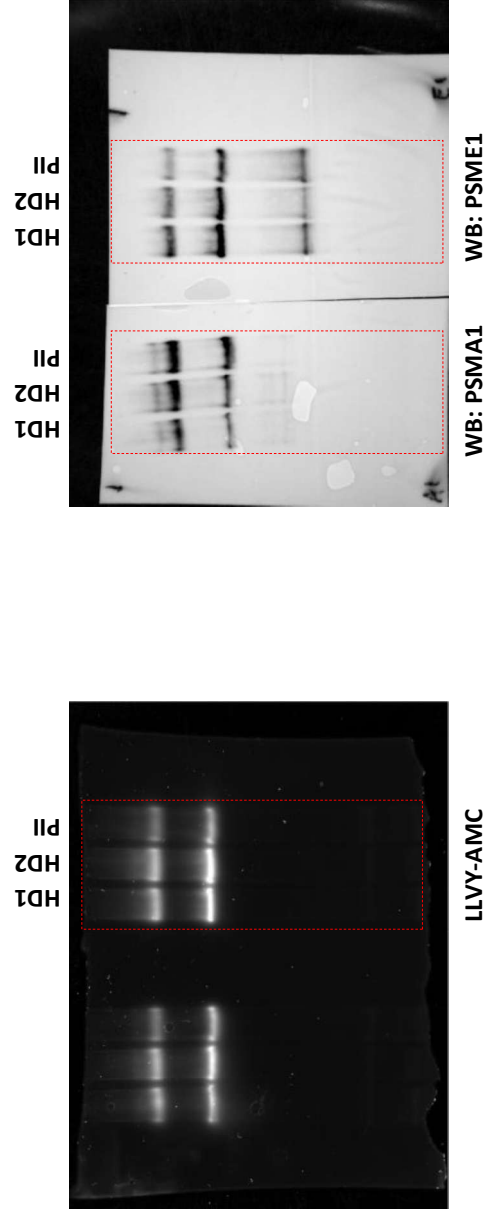

**Fig. 3B**

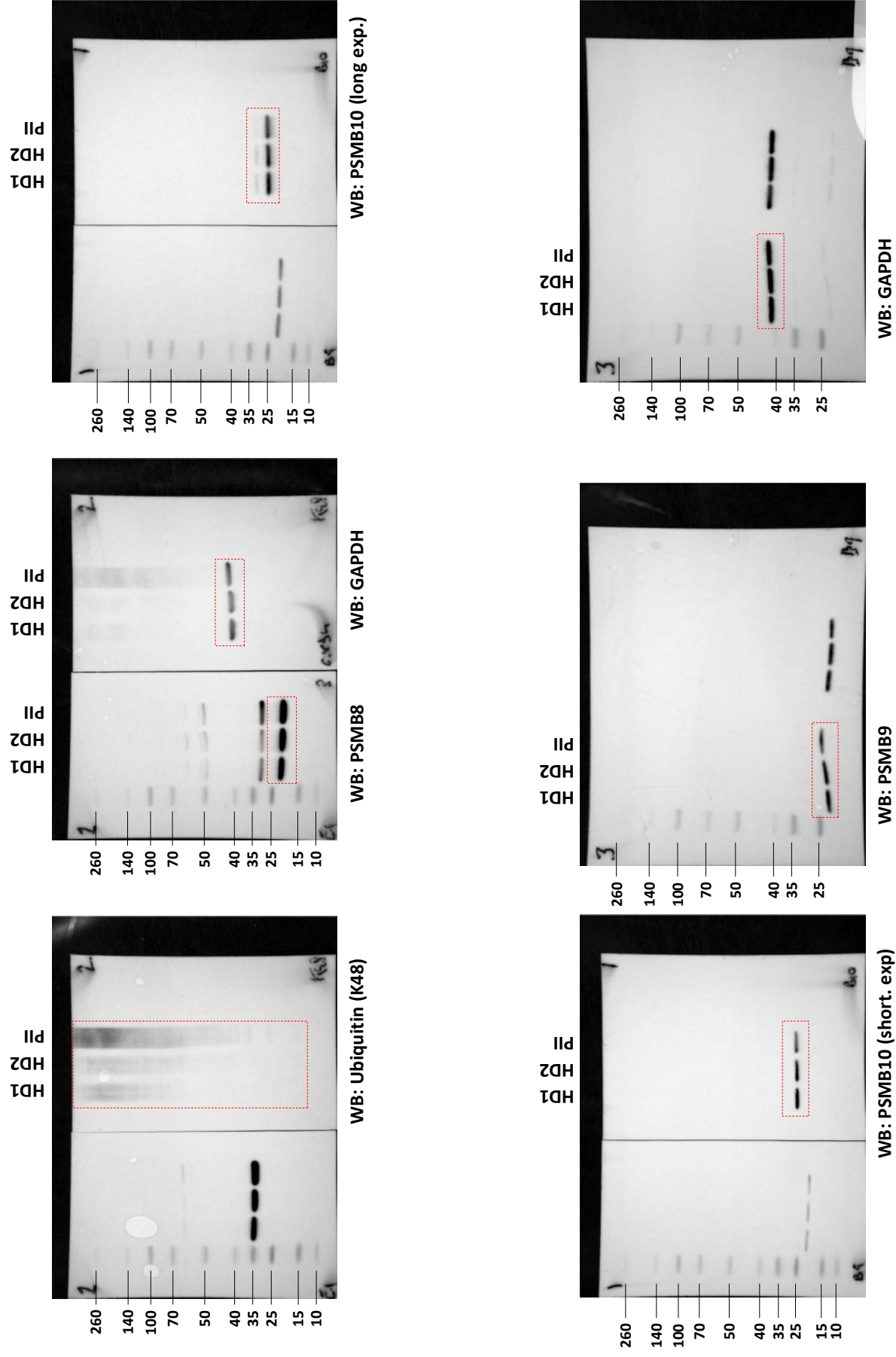

Fig. 3B

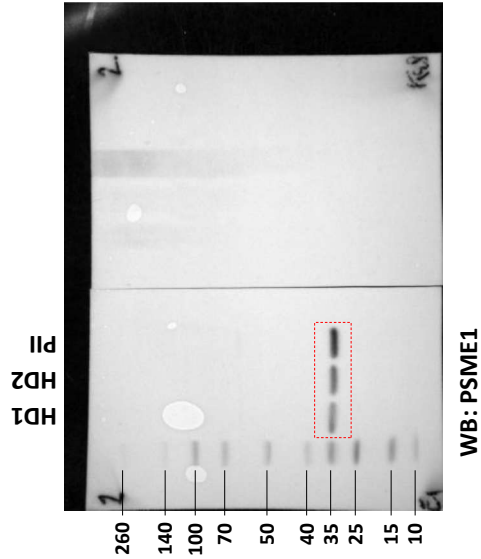

Supplement: SourceData F3 — is the source file for Fig. 3. [file jhi_20250096_sourcedataf3.pdf]

Fig. S1

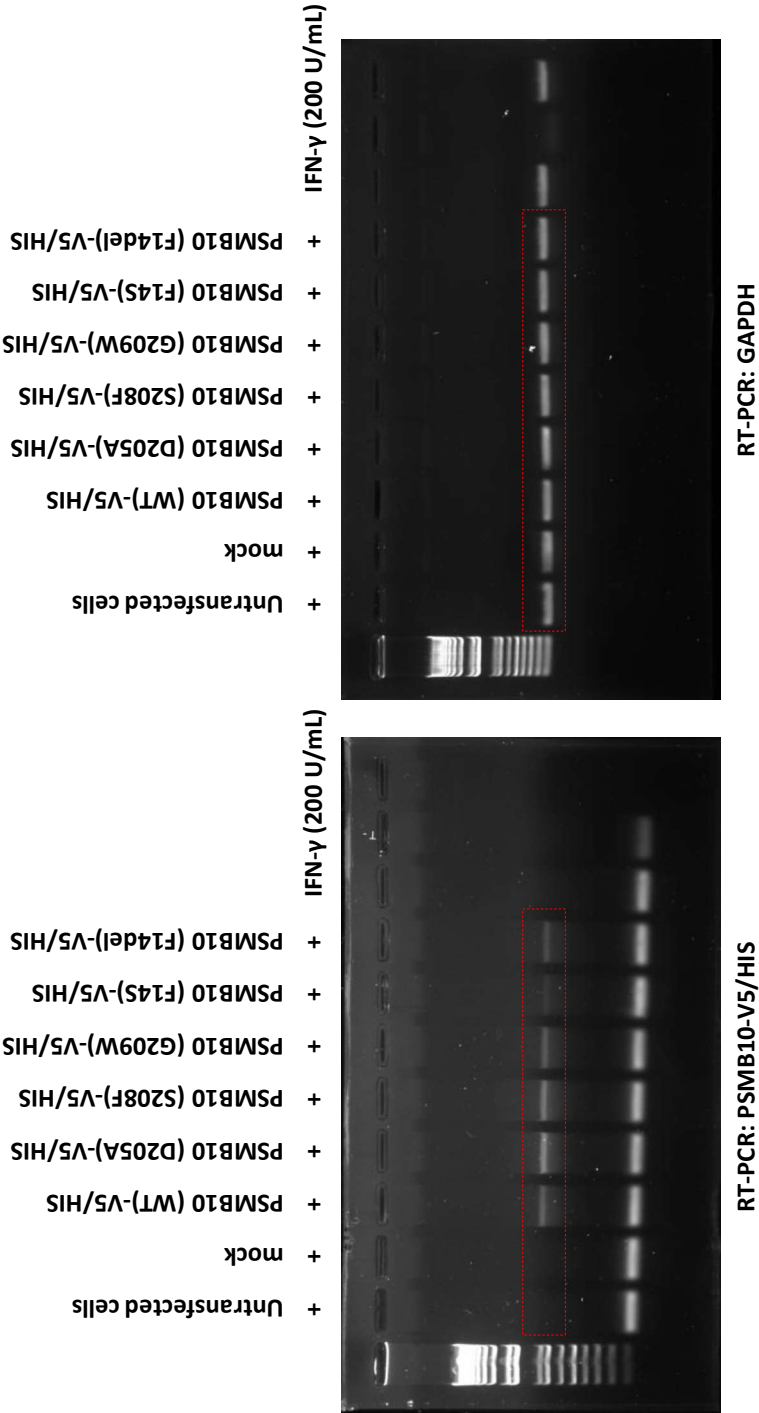

Supplement: SourceData FS1 — is the source file for Fig. S1. [file jhi_20250096_sourcedatafs1.pdf]

Fig. S2

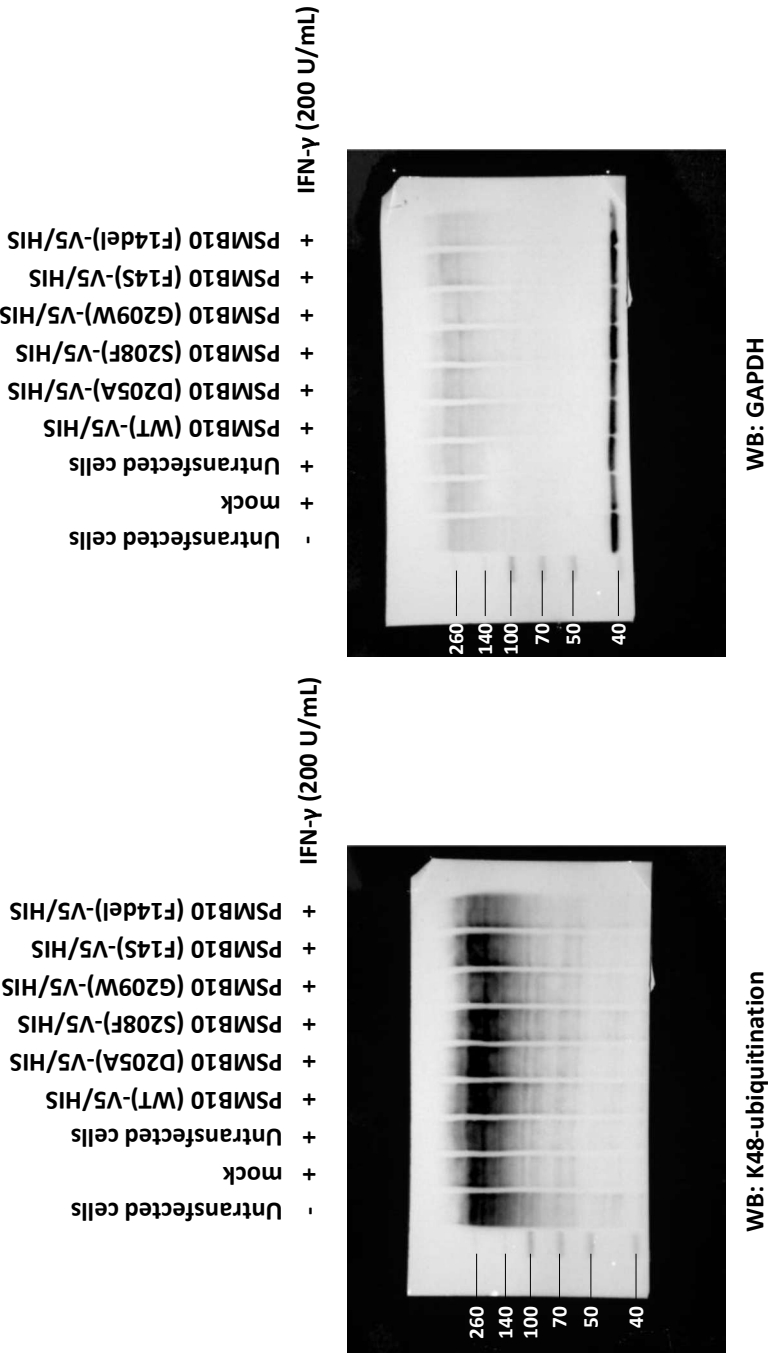

Supplement: SourceData FS2 — is the source file for Fig. S2. [file jhi_20250096_sourcedatafs2.pdf]
